# Ultrasound-Guided Percutaneous Release of A1 Pulley by Using a Needle Knife: A Prospective Study of 41 Cases

**DOI:** 10.3389/fphar.2019.00267

**Published:** 2019-03-26

**Authors:** Min Pan, Shuya Sheng, Zhiqi Fan, Hao Lu, Hong Yang, Fei Yan, Zhansen E

**Affiliations:** ^1^Shenzhen Hospital of Guangzhou University of Chinese Medicine, Shenzhen, China; ^2^Shenzhen Middle School, Paul C. Lauterbur Research Center for Biomedical Imaging, Shenzhen, China; ^3^College of Optoelectronic Engineering of Shenzhen University, SPACEnter Space Science and Technology Institute, Shenzhen, China; ^4^Paul C. Lauterbur Research Center for Biomedical Imaging, Shenzhen Institutes of Advanced Technology (CAS), Shenzhen, China; ^5^Longgang Central Hospital, Shenzhen, China

**Keywords:** ultrasonography-guided, release, A1 pulley, needle knife, trigger finger

## Abstract

**Objective:** The purpose of this study was to evaluate the efficacy of ultrasonography-guided percutaneous A1 pulley release with the needle knife for trigger finger.

**Methods:** The prospective study included 21 patients (21 fingers) who underwent blind release with the needle knife and 20 patients (20 fingers) who underwent ultrasonography-guided release with the needle knife. The thickness and width of A1 pulley, clinical grade before and after release, complications, and operation time were compared between the groups.

**Results:** The results showed that the ultrasonography-guided group had significantly better grade postoperatively and reached to 100% complete release in one time compared to the blind group (*p* < 0.05). Moreover, no any complications had been happened in the ultrasonography-guided group. A relatively longer operation time of the ultrasonography-guided group was observed compared to the time of the blind group.

**Conclusions:** The needle knife is a very good tool for release of triggering fingers. Ultrasound provides a direct and precise visualization of the thickness, width and location of A1 pulley lesion. The combined use of ultrasound and the needle knife can achieve the best result for trigger finger. Moreover, the combination changes the traditional opinion and operator-dependent mode that were once widely adopted in the hospital of Chinese Medicine.

## Introduction

Stenosing tenosynovitis, also called trigger finger (TF), is the snapping and locking of the finger, related mainly to an imbalance between the size of the flexor tendons and that of the tendon sheath (Yin and Guo, 2016; Nikolaou et al., 2017). The cause for TF is thickening of the A1 pulley due to excessive flexion and extension of digits, repeated friction between flexor tendon and tendon sheath, or failure in prompt treatment of palm skin injury. Patients with TF are often diagnosed clinically according to their medical histories, symptoms, and signs. Generally, mild cases are first treated conservatively, with oral anti-inflammatory drugs, physical therapy, or corticosteroid injections; while severe cases are often treated with an open surgical release, which is successful in 83~98% of cases (Paulius and Maguina, 2009).

Blind percutaneous A1 pulley release was first described by Lorthior in 1958 (Paulius and Maguina, 2009). This operation can be done without any special preparation and can obtain the effect equal to that of an open procedure. Besides, this procedure has many advantages, including shorter recovery time, avoidance of scar tenderness, and application in the outpatient setting (Rajeswaran et al., 2009; Rojo-Manaute et al., 2010, 2012a,b; Smith et al., 2010). However, there is still a potential risk of damage to the tendon and neurovascular structures. Also, it is difficult to confirm whether the release is complete or not during operation because of invisualization directly (Lee et al., 2018).

Ultrasound has become widely accepted as an imaging modality in assessment of the musculoskeletal system, as it is quick, cheap, and readily available. Also, it has real-time, non-invasive and non-radiative advantages for musculoskeletal diseases by ultrasonography-guided treatment (Chang et al., 2017, 2018; Wu et al., 2018). To date, ultrasonography-guided percutaneous A1 pulley release has been introduced in this procedure, providing direct visualization of the vascular and nerve structures during the procedure (Hopkins and Sampson, 2014; Hoang et al., 2016; Lapègue et al., 2016; Rajeswaran et al., 2016; Guo et al., 2017). With the application of high-frequency ultrasonographic instrument, the flexor digitorum tendons, pulley systems, volar plate, metacarpophalangeal and interphalangeal joints can be clearly seen. Moreover, the TF pathologic anatomic structures identified by ultrasound are even far superior by MRI, especially in the dynamic evaluation. The ultrasonographic characteristics of TF are hypoechonic thickening of the A1 pulley, or increased Doppler flow and the fluid of surrounding tissues.

To facilitate surgeon manipulation, some simple clinical tools are developed for the A1 pulley surgical release, such as a 19 gauge needle or 21 gauge needle (Hoang et al., 2016; Lapègue et al., 2016; Rajeswaran et al., 2016; Guo et al., 2017). Lapègue et al. (2016) reported US-guided percutaneous release of the TF by using a 21-gauge needle, achieving an 81.7% complete resolved cases immediately after the procedure with minimal complications. However, it was reported that the gauge needles can be twisted easily and the sharp tip might increase the possibility of hurting the surrounding tissues. Specially designed knives have been used for percutaneous release, including the knife with a hook shape or with long body (Nikolaou et al., 2017; Lee et al., 2018). A success rate of 100% using this knife was achieved in ultrasonography-guided percutaneous release. However, the long body makes it difficult to control and the hook shape might hurt the tendon during operation. Moreover, the designed knife is not common everywhere.

The needle knife is a traditional tool of Chinese medicine which has been used widely by the rehabilitation doctors of China since thousands of years ago. TF is the preferred alternative for the needle knife (Ma and Wu, 2016). However, blind percutaneous release has several native problems. For example, inexperience of the operator and anatomical variation of the patient can lead to accidental injury to the flexor tendon or adjacent neurovascular bundles, as well as difficulty in determining the completeness of release. In addition, a vertical insertion from above the skin surface may increase the risk of nerve and the flexor tendon damage (Ma and Wu, 2016; Lee et al., 2018).

In this study, we attempted to evaluate the efficacy of ultrasonography-guided percutaneous A1 pulley release with the needle knife.

## Materials and Methods

Our clinical study was approved by the Research Ethics Committee of Shenzhen Hospital (Futian) of Guangzhou University of Chinese Medicine. All patients were conducted by a rehabilitation doctor (Shaoyang Cui, 10 years of experience) and an interventional radiologist specializing in musculoskeletal ultrasound (Min Pan, 15 years of experience).

### Study Population (Figure 1)

During a 12-month period starting in March 2017, 97 patients were enrolled in our prospective study. The range of age was from 45 to 72 years (average age 57 ± 8 years), with trigger finger, Grade II-IV.

**Figure 1:**
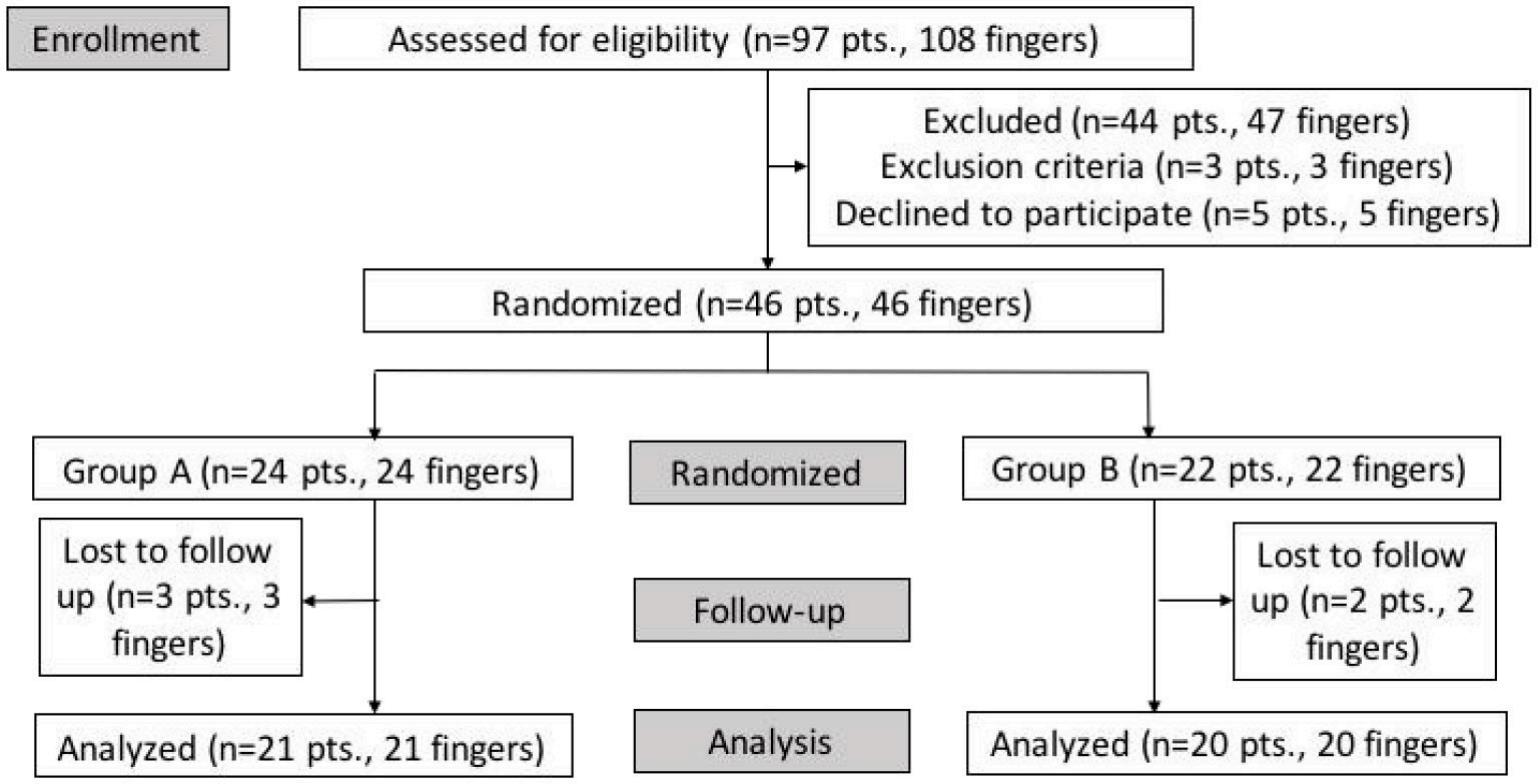
Patients' flowchart.

The inclusion criterion was idiopathic trigger finger present for at least 3 months. The exclusion criteria were a previous history of open release for trigger finger, rheumatoid arthritis, a concomitant pathologic condition in hand at the first visit to the rehabilitation doctor, and A1 pulley thickness more than one finger.

After being diagnosed and graded by the Department of Rehabilitation, the patients were required to write informed consent at the first visit. Of 97 patients (108 fingers), 44 patients (47 fingers) were excluded due to an improvement in symptoms after conservative treatments or triggering more than one finger. And five patients (5 fingers) refused to participate in the clinical research. Three patients (3 fingers) were excluded due to rheumatic arthritis. Finally, a total of 46 patients (46 fingers) were included in this study. Patients were divided into two groups randomly. Twenty-four patients (24 fingers) included in group A underwent blind percutaneous A1 pulley release, while 22 patients (22 fingers) included in group B underwent ultrasonography-guided percutaneous A1 pulley release. A total of five patients were lost in the last follow-up. Thus, 21 patients (21 fingers) in group A and 20 patients (20 fingers) in group B were analyzed (Table 1).

**Table 1:** Demographics of patients^*^.

	**Group A (Blind release)**	**Group B (Ultrasonography-guided release)**
Number of patients	21	20
Mean age (years)	56 ± 6 (47–67)	58 ± 10 (45–72)
Sex	F	F
Mean follow-up (weeks)	15.7 (15–18)	12.2 (12–13)
Digit involved Thumb/index/middle /ring/small	6/3/9/3/0	7/2/8/3/0
Thickness of A1 pulley by US (mm)	1.80 ± 0.44 (1.20–2.40)	1.49 ± 0.23 (1.10–1.80)
Width of A1 pulley by US (mm)	5.59 ± 0.76 (4.40–7.10)	5.29 ± 1.16 (5.00–6.30)

* *No significant differences between two groups*.

### The Clinical Diagnostic Criteria of TF

(1) The history of finger microtrauma or overuse; (2) Pain, tenderness, or palpable nodules at the proximal palmar crease; (3) Limited finger flexion and extension; (4) Positive value of flexor resistance test.

According to the degree of entrapment between the flexor digitorum tendon and tendon sheath, TF are divided into five grades (Lapègue et al., 2016; Lee et al., 2018) (clinical semi-quantitative evaluation criteria): (1) Grade 0: no triggering; (2) Grade 1: intermittent, moderate triggering; (3) Grade 2: continuous triggering that is eliminated with active extension; (4) Grade 3: triggering with flexion contracture that requires the patient to use the other hand to unlock the involved finger; (5) Grade 4: active flexion of finger is impossible.

### Ultrasonic Examination

Aplio 500 (Toshiba company, linear array probe PLT-1005BT, frequency 5~14 MHz) and Resona 7 (Mindray company, linear array probe L14-5WU, frequency 6.6~14 MHz) ultrasound imaging machines were used.

The patients received ultrasound examine before release, day 0 and day 7 after release. Patients sat on the chair with palm up on the bed. The probe was placed on the metacarpophalangeal joints. The short and longitudinal axes were observed along the tender point and/or painful nodules. The dynamic examine was carried out as the flexion and extension of finger (see Videos 3, 4). The thickened A1 pulley was measured and marked by ultrasound: (a) The thickness of A1 pulley in short axis. (b) The length of the thickened A1 pulley in longitudinal axis.

### The Procedure of Release

**Figure 2:**
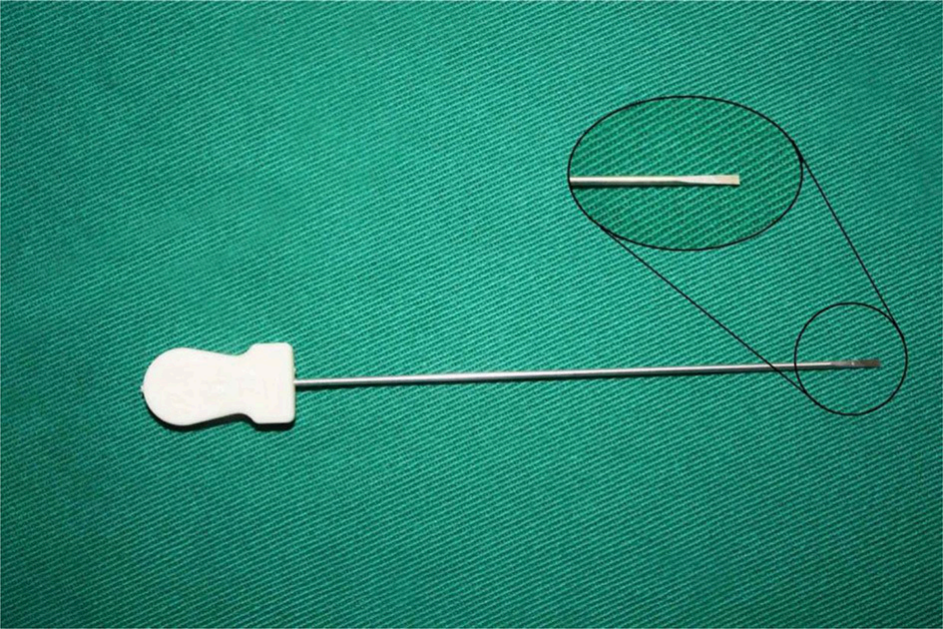
The scheme of Hanzhang needle knife. The needle knife consists of three parts: tip, body and handle, with a 0.8 mm blade on the tip, and 40 mm length of the body. The tip can serve as a scalpel during release.

Hanzhang needle knife (Figure 2, Beijing Huaxia Needle Knife Medical Equipment Factory) was used. All patients were treated with only needle knife, and no any other treatment such as local injection was used. The dynamic flexion and extension of fingers before and after release were recorded immediately (see Video 2). All patients were graded again by the semi-quantitative evaluation in day 0 and day 7 after release.

**Figure 3:**
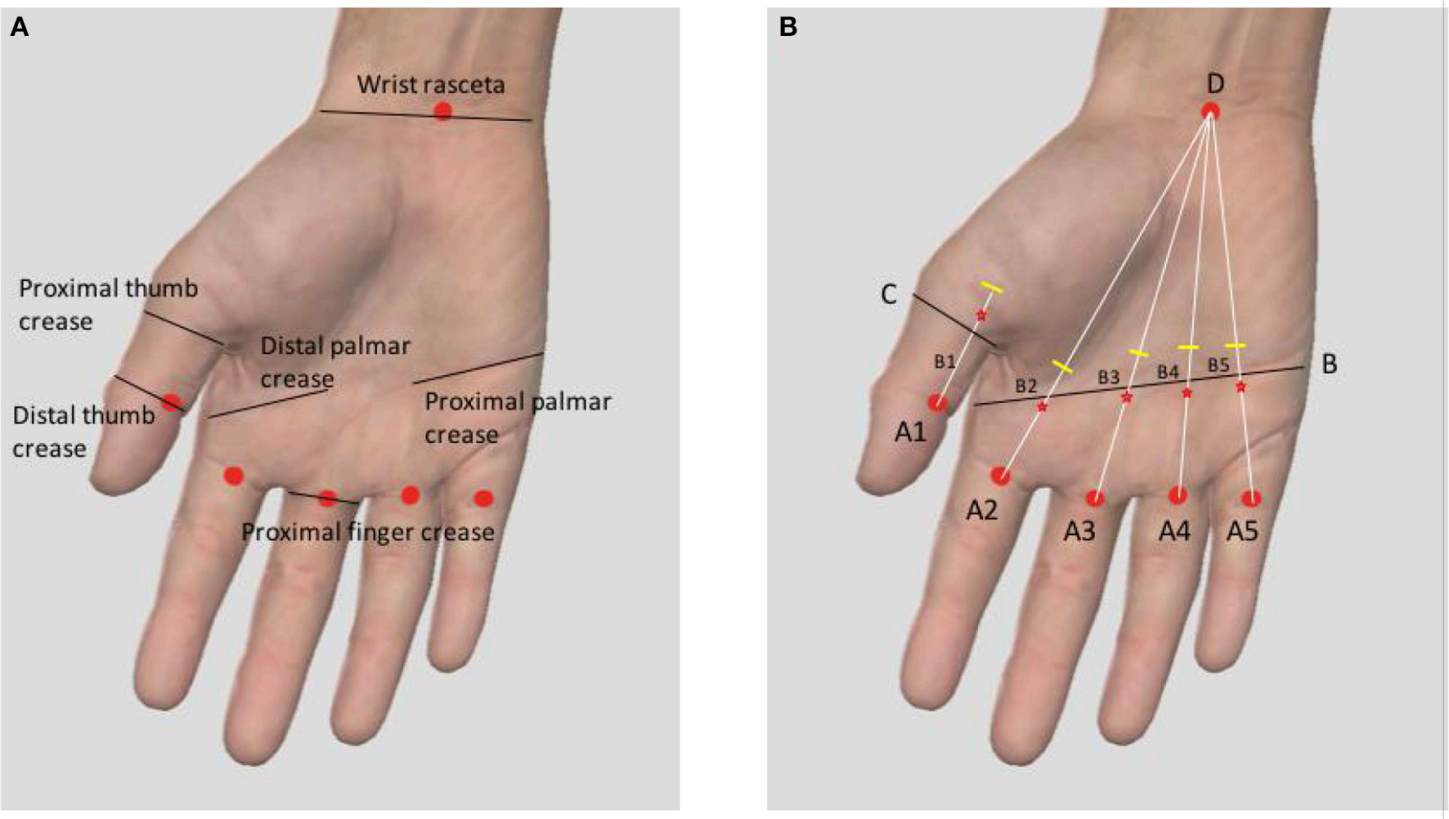
**(A)** The anatomical landmark of entry point. **(B)** The marking method of the entry point (Triggering thumb: A straight line paralleling to the thumb from the midpoint A1 of the distal thumb crease is drawn. The line C is the transverse line of proximal thumb crease. B1 is the intersection point of A1 parallel line and C line. The entry point is +0.5 mm of B1 proximally. Triggering finger: A connection line is drawn between the midpoint of the proximal finger crease (A2~A5) and D (the midpoint of wrist rasceta). Line B is drawn between the distal and proximal palmar creases. B2~B5 are the intersection points of lines B and line AD. The entry point is −0.5 mm of B2~B5 distally).

**Figure 4:**
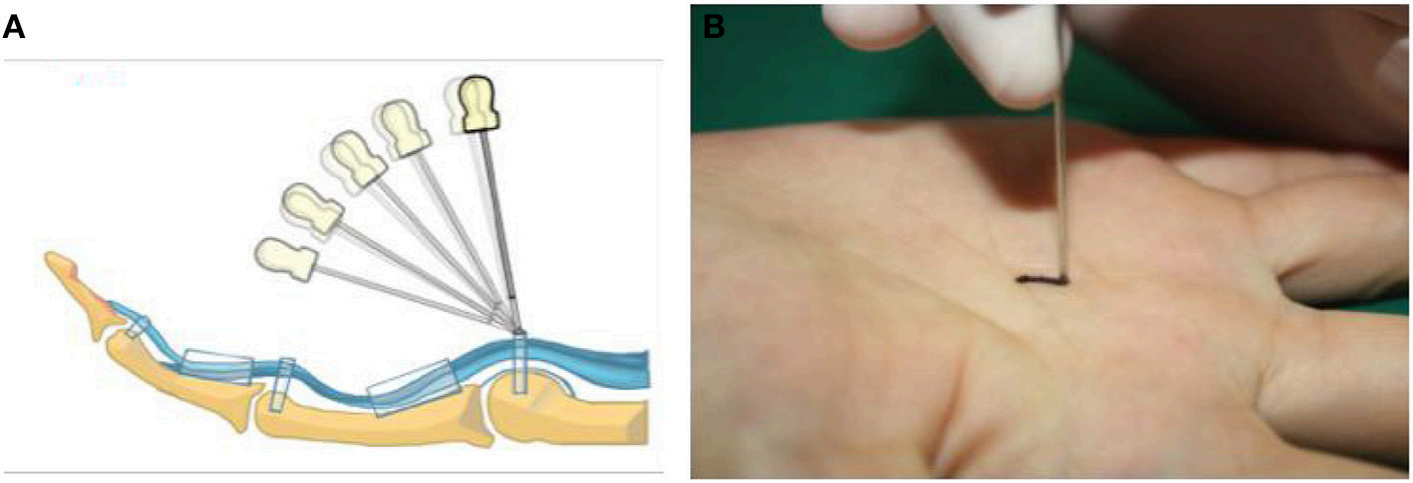
The scheme **(A)** and spot **(B)** of the blind release.

The group A followed three steps: (1) Fix point (Figure 3): The point was set proximally to avoid the painful nodule according to the anatomical landmarks and the marking entry point. (2) Fix orientation (Figure 4): The body of the needle knife was perpendicular to the skin surface. The blade-edge line is parallel to the imaginary line of flexor tendon sheath; (3) Stab: The tip of the needle knife was stabbed into the skin quickly, and then entered into sheath slowly and carefully. For an operator, if a break is felt, he should stop piercing further, and start to cut 1.0~1.5 mm proximally. Meanwhile, the handle of the needle knife is slightly tilted distally, shifting from an angle of 90° to 60° to 30° on the skin surface (Figure 4A). The total forward cutting number is five to six. In backward direction, five to six cuts are performed again along the marking orientation (A1 parallel line of thumb or a connection line AD of finger).

The group B were monitored by ultrasound during the whole procedure. The tip and body of the needle knife were parallel to the tendon (see Video 1) and (Figures 6A,B).

### Statistical Analysis

The descriptive data were tested for normal distribution. Differences in clinical outcome were analyzed using the Student's *t*-test. A *p* < 0.05 was considered statistically significant. Data were expressed as mean ± standard deviation.

## Results

Age, mean follow-up, involvement of digit, and the thickness and width of A1 pulley were evaluated as demographic factors. No significant differences were found in demographic characteristics between two groups (Table 1).

No significant difference was found in the clinical grade of two groups before release (Table 2). Almost all patients showed significant improvement in clinical grade after release (*p* < 0.05). In addition, the group B (the ultrasonography-guided group; Figures 7A–C). showed significantly better grade at day 0 and day 7 postoperatively compared with the group A (the blind group, *p* < 0.05).

**Table 2:** Type of Trigger Finger before Release, Day 0 and Day 7 after Release (*n* = 41).

**Grade and type of trigger finger**	**Group A (*****n*** **= 21)**			**Group B (*****n*** **= 20)**		
	**Before release**	**Day 0 after release**	**Day 7 after release**	**Before release**	**Day 0 after release**	**Day 7 after release**
Grade 0	0 (0)	0 (0)	4 (19.0)	0 (0)	11 (52.4)	20 (100.0)
Grade 1	0 (0)	16 (76.1)	15 (71.4)	0 (0)	8 (40.0)	0 (0)
Grade 2	2 (9.5)	3 (14.2)	0 (0)	2 (10.0)	1 (5.0)	0 (0)
Grade 3	8 (38.0)	1 (4.8)	1 (4.8)	10 (50.0)	0 (0)	0 (0)
Grade 4	11 (52.4)	1 (4.8)	1 (4.8)	8 (40.0)	0 (0)	0 (0)

*Data in parentheses are percentages*.

**Figure 5:**
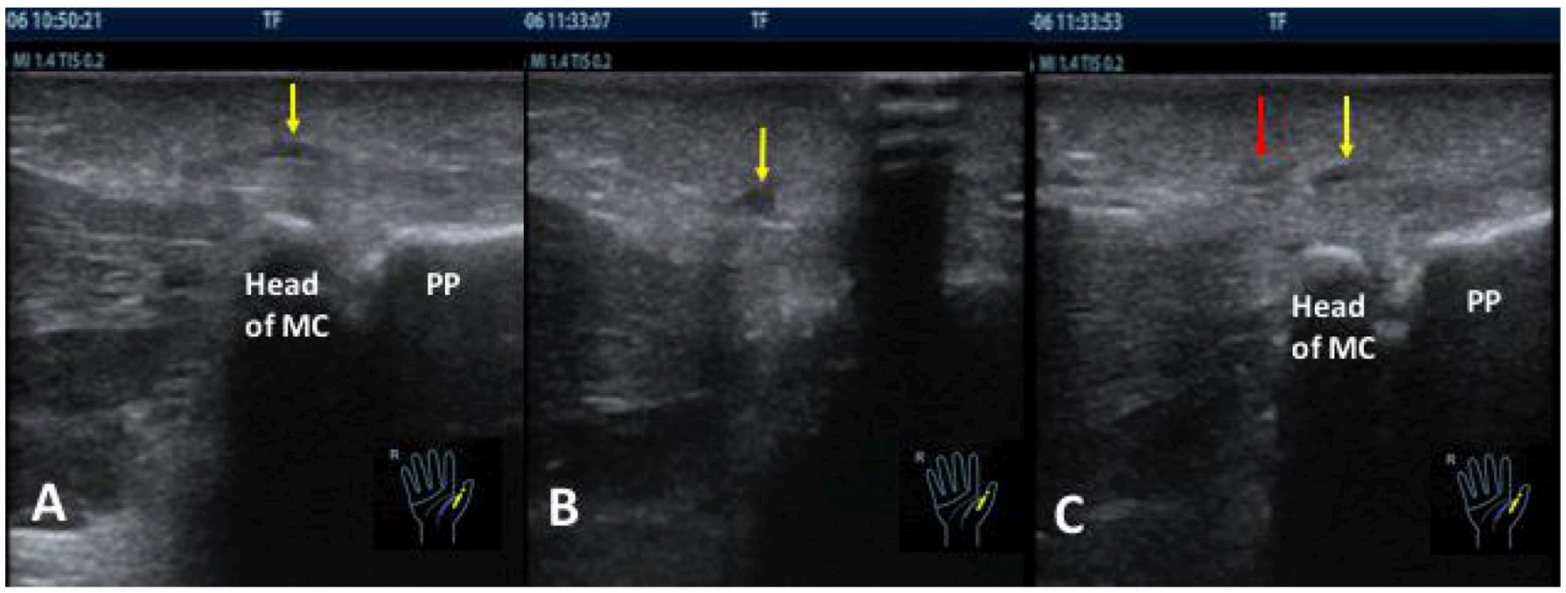
The ultrasound images before **(A)** and after **(B,C)** release in the blind group. **(A)** The yellow arrow showed the thickness of A1 pulley in the right thumb before release. **(B)** The yellow arrow showed the fluid of the surrounding tissue immediately after release. **(C)** The red arrow was the wrong cutting direction after piercing into the skin from the marked entry point. The yellow arrow was the thickening location of A1 pulley. They were not at the same point. PP, proximal phalange, MC, metacarpal bone.

Triggering disappeared in all patients who underwent ultrasonography-guided release, whereas mild triggering continued in 15 patients who underwent blind release at day 7. In 1 case of group A, no significant improvement was found in clinical grade before release, day 0 and day 7 after release. In one case of group A, the blade of the needle knife was deviated from the A1 pulley after incision, and the fluid of surrounding tissue was found immediately after release (Figures 5A–C). Ultrasonography-guided release was performed in these two patient at 4 weeks postoperatively.

**Figure 6:**
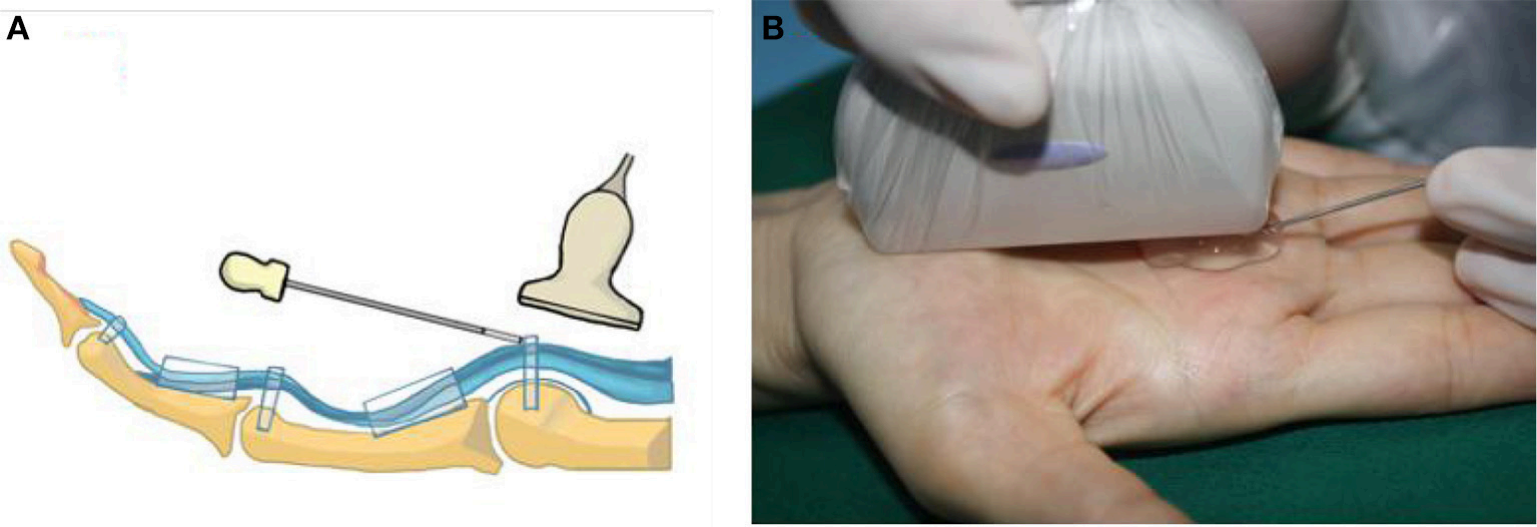
The scheme **(A)** and spot **(B)** of the ultrasonography-guided release. The body of the needle knife paralleled to the tendon. With the help of ultrasound, it was easy and safe to complete the whole procedure.

**Figure 7:**
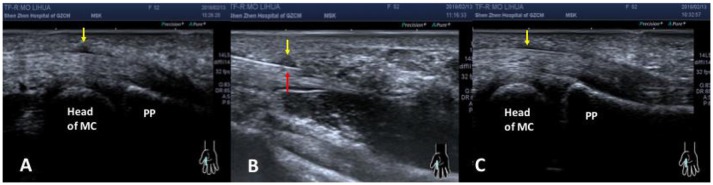
The ultrasound images before **(A)**, during **(B)** and after **(C)** release with ultrasonography-guided. **(A)** The yellow arrow showed the thickening of A1 pulley in the right index finger before operation. **(B)** The red arrow showed the needle knife was cutting the A1 pulley (yellow arrow). **(C)** The A1 pulley (the yellow arrow) became normal thickness immediately after ultrasonography-guided release. PP, proximal phalange; MC, metacarpal bone.

A relatively longer operation time of the ultrasonography-guided group (15.21 ± 0.87 min) was observed compared to the time of the blind group (5.23 ± 0.55 min, *p* < 0.05).

## Discussion

Flexor digitorum tendon sheath extends distally from the metacarpal neck to the distal interphalangeal joint. The tendon fiber sheath thickens in different areas to form a series of dense connective tissue bundles with different widths, thicknesses, and morphologies. This structure is called the flexor tendon sheath pulley system, which consists of five annular pulleys (A1~A5), four cruciform pulleys (C1~C4), and one palmar aponeurosis pulley. The A1 pulley is attached to the volar plate of the metacarpophalangeal joint, with an average width of 7.1 mm, and an average thickness of <1 mm.

It has not been very clear which is earlier predominant factor of triggering finger considering the injured pulley or tendinopathy, but both are involved when clinical symptom appears. During the early stage, aseptic inflammations such as hemorrhage, edema occur around the tendon sheath. At a later stage, the chronic pathologies of pulley and tendon such as hypertrophy, adhesion occur. The thickness of pulley can increase up to 2~3 mm from the normal value, which is <1 mm. The annular stenosis forms as the pulley system at the lesion thickens. Patients suffer from dysfunction of flexion and extension of fingers, and this situation is particularly obvious when waking up early in the morning. A feeling of bounce occurs at the nodule when fingers are flexed and extended, and the subcutaneous nodular-like lump is palpable. In the early stage, the flexor tendon slides over the stenosis of the pulley with difficulty, resulting in a trigger-like movement. In the later stage, patients cannot flex or extend actively, keeping in a stiff position. This condition is called “locking and snapping.”

The needle knife is a traditional tool of Chinese medicine based on the therapy of “damage first, recover later.” It consists of three parts: tip, body and handle. It has a 0.8 mm blade on the tip, and its length is 40 mm. It does not need to cut the skin to enter the body and reach the lesion. The main role of a needle knife is to loosen adhesion that improves blood circulation, increases the metabolism of local pain-causing substances, and relieves tension.

Blind needle knife release by using palm anatomical landmarks has been well-known as the rehabilitation doctors of China since thousands of years ago (Paulius and Maguina, 2009; Yin and Guo, 2016). The effectiveness has been accepted by clinical doctors and patients. TF is the preferred alternative for the needle knife. Other illnesses, such as radial styloid stenosing tenosynovitis, carpal tunnel syndrome, ankle tunnel syndrome, ganglion cyst, chronic fasciitis, and trigger point release are often treated by the needle knife. The operator's experience during the operation of the needle knife is the key to determine if release is completed, flexion and extension is recovered, and the surrounding tissue is damaged. Thus, the complications such as damage to interdigital nerves, vessels, or flexor tendon have been existed. On the other hand, it is common of the recurrence and incomplete release, or rare tendon rupture due to the repeated steroidal injection inaccurately.

The result showed that almost all patients had significant improvement after release. However, comparing to the ultrasonography-guided group in which the completion of release was 100% in all patients at day 7 postoperatively, that of the blind group was 19.0%, and mild triggering of the blind group was 71.4% postoperatively. In the blind group, one case had such complications as the fluid of surrounding tissue and cutting in the wrong place, and one case had no improvement. Both were received ultrasonography-guided release 4 weeks later after their first blind releases. The causes for limited efficiency in the blind group were: (1) The process of the cutting diverged from the lesion. Figure 5C showed that the cutting position after piercing into the skin from the marked point was not the same as the location of thickening pulley. (2) The cutting depth was difficult to control. If cutting is too superficial, the snapping cannot be released. Likewise, if it is too deep, it is possible to injure the tendon or surrounding tissue. Figure 5B showed the fluid of surrounding tissue. (3) The cutting width was difficult to control: The tip of the needle knife has a blade of 0.8 mm. Generally, five to six cuts proximally and distally were performed along the imaginary line of flexor tendon sheath with a cutting width of 4~4.8 mm. The range of thickening width of A1 pulley measured by ultrasound before release in the blind group was 4.40~7.10 mm. It meant that there was an incomplete cut existed partly in the blind group.

## Conclusion

The needle knife is a very good tool for release of triggering fingers. The combined use of ultrasound and the needle knife can achieve the best result for trigger finger.

Ultrasonography-guided release of trigger finger with the needle knife is feasible and safe in current clinical practice. With the help of ultrasound, it will be independent of operator's experience, easy to solve the problems of depth and width in the cutting process, or the injury of surrounding tissue.

Complete one-time release of trigger finger was achieved in all ultrasonography-guided release in 1 week.

Our microinvasive procedure is nearly painless and requires less than half a day off work for all of our subjects.

## Limitation

There were several limitations in our study, firstly that this was not a randomized controlled trial as the development stage of each patient was different. Secondly, the age of patients in our study was concentrated among middle aged and elderly people, and the gender of that was female, no male. Thirdly, the follow-up time of patients postoperatively was short. The long-term recovery value of ultrasound-guided release in the patients of trigger finger still needs to be further explored through prolonged follow-up time. Finally, there was a relatively longer operation time of the ultrasonography-guided group compared to the time of the blind group. Time will decrease if more practice and collaboration are maintained between the rehabilitation doctor and ultrasound doctor.

## Data Availability

All datasets generated for this study are included in the manuscript and/or the Supplementary Files.

## Author Contributions

MP and SS carried out analysis and calculations of data, drawings, and contributed to the writing of the manuscript. HL and HY contributed to data collection and management. ZF contributed to the release by needle knife. FY and EZ designed and coordinated the project, contributed to the discussion, supervised and reviewed the writing of the manuscript. All authors read and approved the final manuscript.

### Conflict of Interest Statement

The authors declare that the research was conducted in the absence of any commercial or financial relationships that could be construed as a potential conflict of interest.
